# Supplementary material for: A new electrode design for ambipolar injection in organic semiconductors
Source: Nat Commun. 2017 Oct 17;8:999. doi: 10.1038/s41467-017-01047-9 (PMC5645469; doi:10.1038/s41467-017-01047-9)
Supplement: Supplementary file 1 — Supplementary Information [file 41467_2017_1047_MOESM1_ESM.pdf]

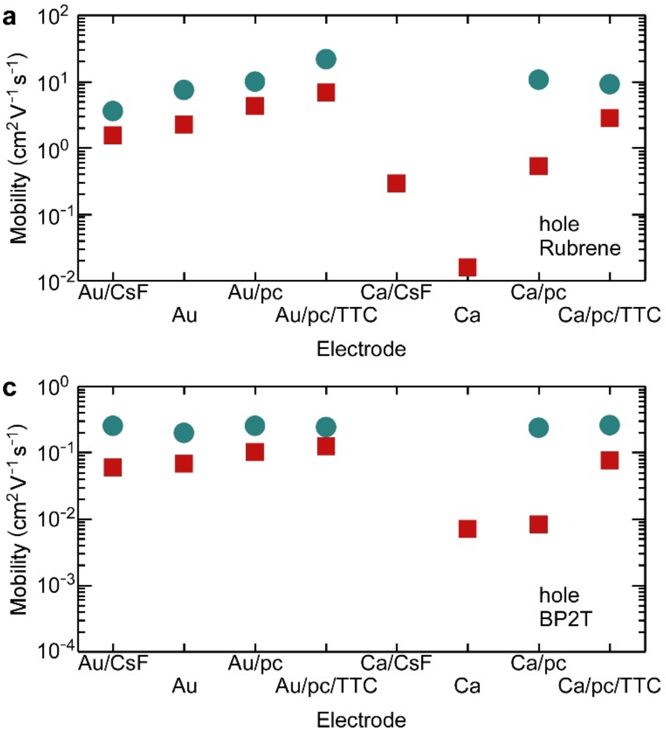

Supplementary Fig. 1 Four-terminal (cyan circles) and two-terminal (red squares) mobilities of single-crystalline organic semiconductor (sc-OSC) field-effect transistors with M/CsF, M, M/polycrystalline-OSC (pc-OSC) and M/pc-OSC/ tetratetracontane (TTC) electrode (M = Au or Ca). **a**, **b** P-type and n-type mobilities of rubrene (RU). **c**, **d** P-type and n-type mobilities of 5,5'-di(4-biphenylyl)-2,2'-bithiophene (BP2T). Reliable values were not obtained for some transistors because of large contact resistance. The mobilities were obtained from transfer curves with drain voltage of 100 V measured at room temperature.

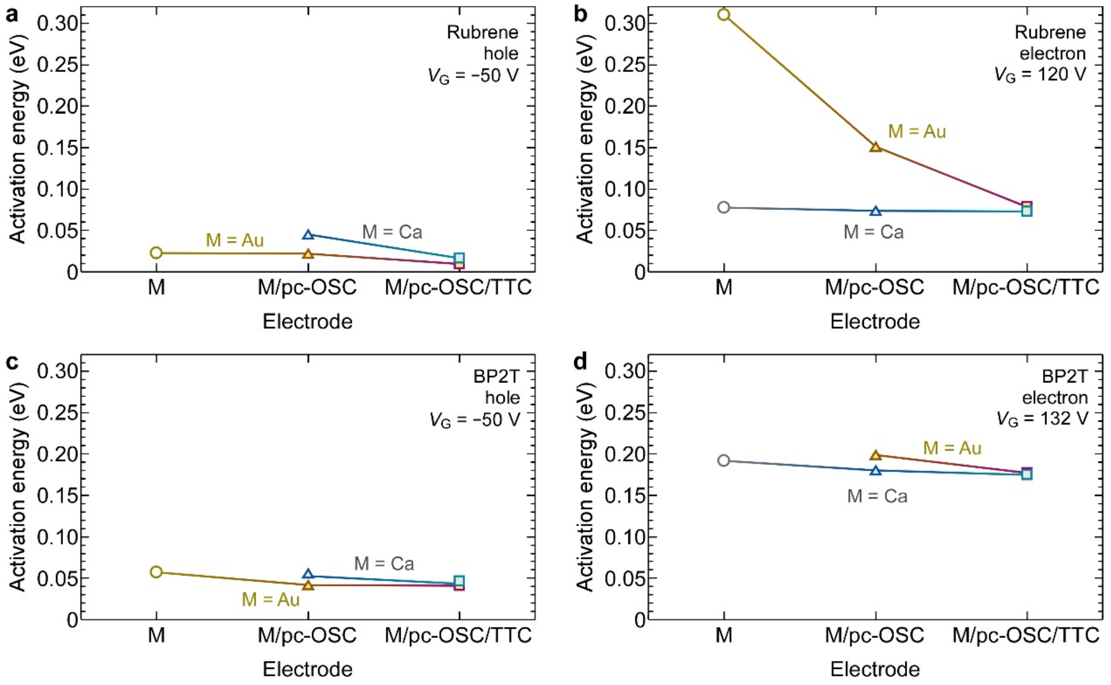

Supplementary Fig. 2 Activation energies for carrier injection in single-crystalline organic semiconductor (sc-OSC) field-effect transistors with M, M/pc-OSC and M/pc-OSC/ tetratetracontane (TTC) electrode (M = Au or Ca). **a**, **b** Hole and electron injection into a RU single crystal. **c**, **d** Hole and electron injection into a RU single crystal. The activation energies were obtained from Arrhenius fittings of the contact resistances measured from 300 to 230 K. The temperature was controlled with a Peltier device. Activation energies of Ca electrode for hole injection and Au electrode for electron injection into BP2T could not be obtained because of very large contact resistances.

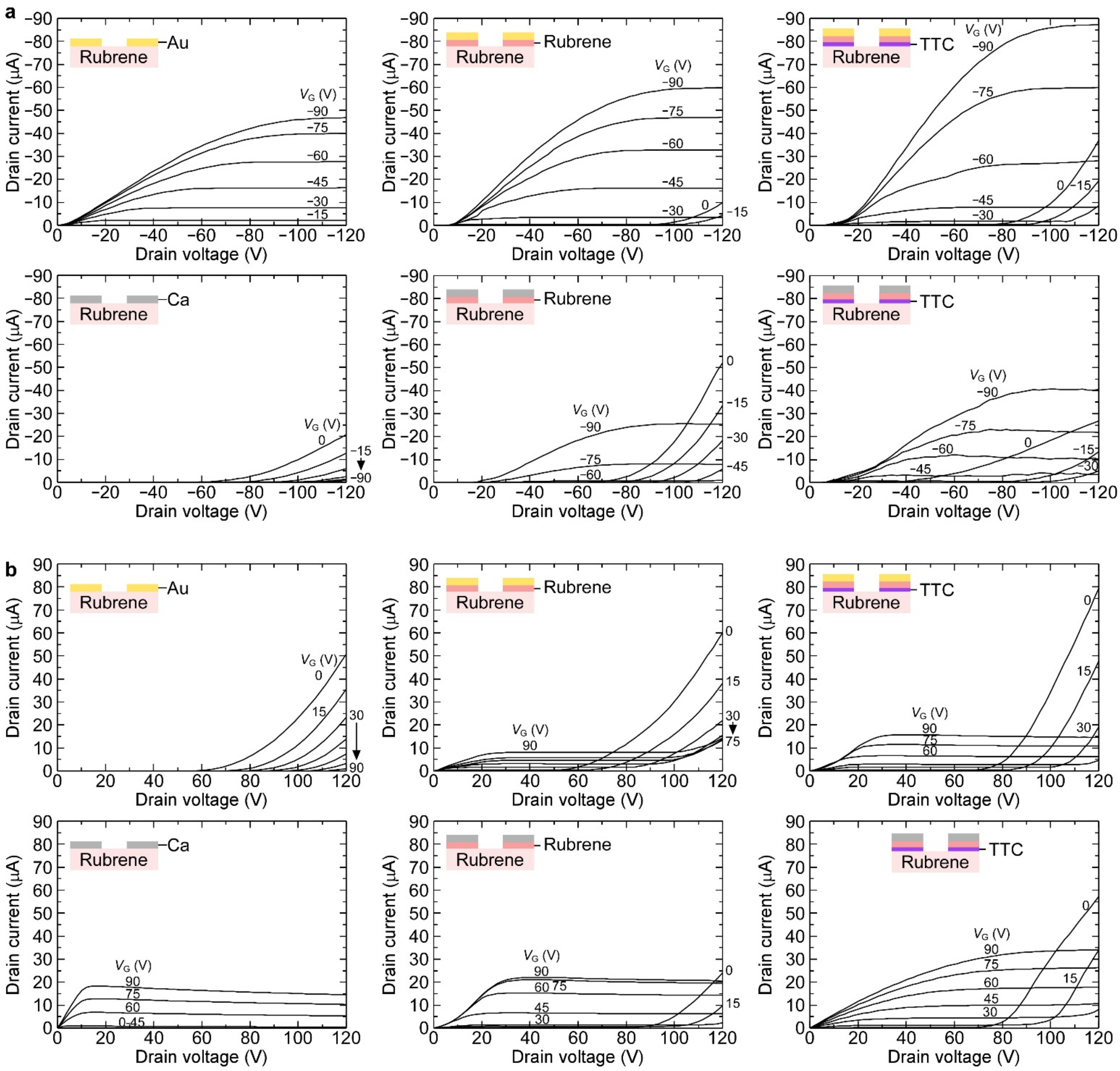

Supplementary Fig. 3 Output characteristics of sc-RU field-effect transistors with M, M/pc-RU and M/pc-RU/tetratetracontane (TTC) electrode (M = Au or Ca). **a** P-type characteristics. **b** N-type characteristics. Left panels: M electrode. Center panels: M/pc-RU electrode. Right panels: M/pc-RU/TTC electrode. Upper panels: M = Au. Lower panels: M = Ca.

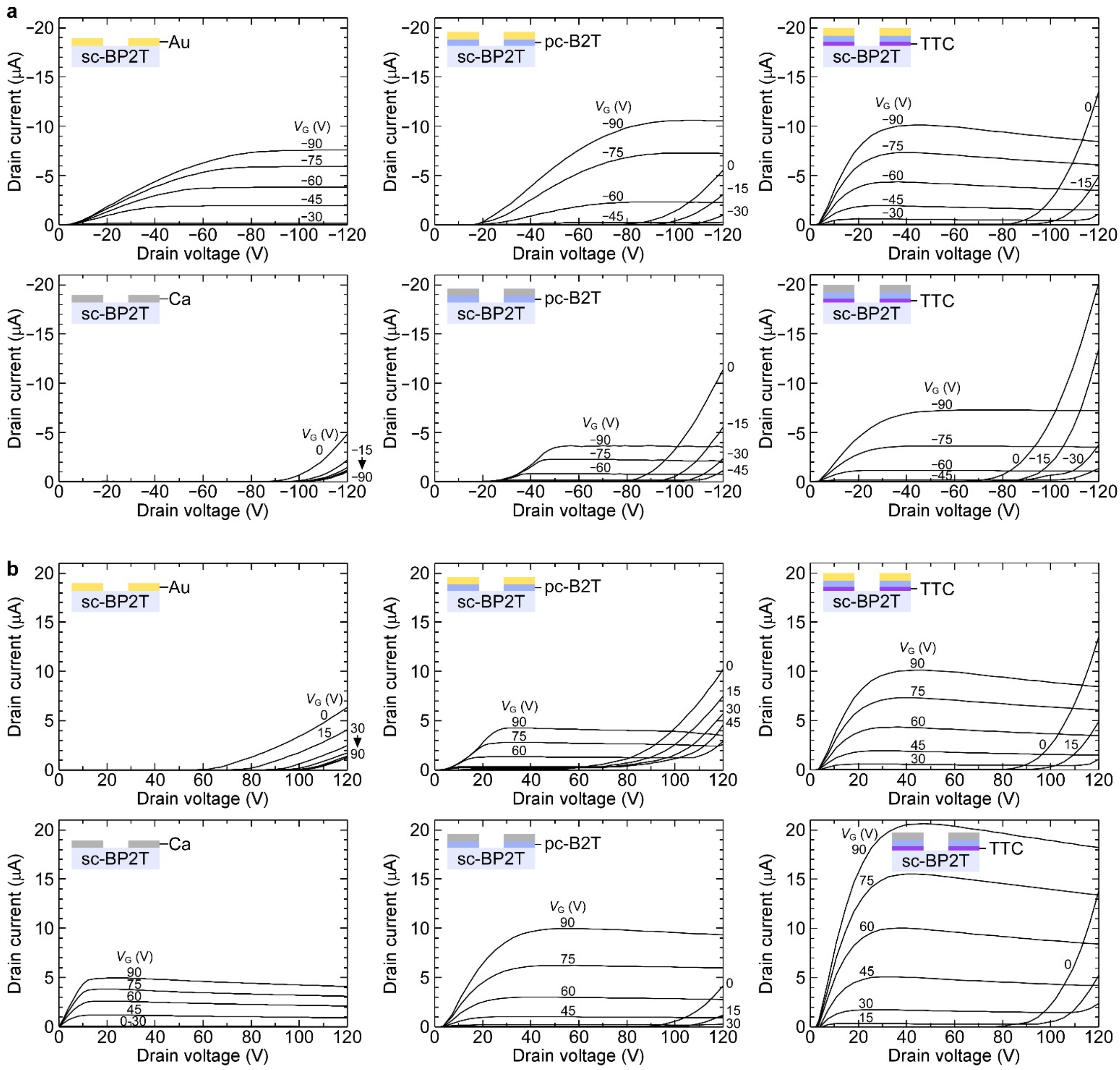

Supplementary Fig. 4 Output characteristics of single-crystalline 5,5′-di(4-biphenylyl)-2,2′-bithiophene (sc-BP2T) field-effect transistors with M, M/pc-BP2T and M/pc-BP2T/ tetratetracontane (TTC) electrode (M = Au or Ca). **a** P-type characteristics. **b** N-type characteristics. Left panels: M electrode. Center panels: M/pc-BP2T electrode. Right panels: M/pc- BP2T /TTC electrode. Upper panels: M = Au. Lower panels: M = Ca.
